# On the Casualties Occurring in the Cooling Treatment of Gout

**Published:** 1805-05-01

**Authors:** James Mantal

**Affiliations:** Bromley High Elms


### On the Casualties occurring in the cooling Treatment of Gout.

To the Editors of the Medical and Physical Journal.

Gentlemen,

SINCE last I had the honour of adressing you on the subject of gout, the Medical and Physical Journal, a work as much distinguished for its impartiality in the selection of the materials from which it is composed, as it is eminently calculated for the diffusion of medical science, in all its branches, contains two interesting cases; one communicated by Messrs. Scott and Taynton, and the other by Mr. O'Neal; both of them so dehortatory from the refrigerant treatment of gout, provided *a due degree of caution* be not used in its adoption, that I cannot but feel myself strongly inclined to offer a few remarks on each of those cases. My intention principally is, to deduce from them the propriety, and even the necessity, of being guided by some *principle*, in the administration of so powerful a remedy; and besides, erecting the superstructure of any doctrine on the foundation of cases detailed by others, is the likeliest method, I conceive, of obviating every suspicion of

 being being biassed in my opinion by a preconceived theory. Notwithstanding the *confidence* with which the refrigerant treatment is recommended by Dr. Kinglake, in all cases indiscriminately, the *arguments* by which he has supported his doctrine, and the *experiments* by which it is sanctioned, it is, I think, sufficiently apparent, from well-attested instances of failure, where his plan of cure has been acted upon, that his theory, in some particulars, stands in need of reconsideration; and that the practice, which, at the outset, promised such immediate and complete relief to a most excruciating malady, is, for want of such revision, in danger of falling into undeserved disrepute and consequent disuse; and for no other reason, than on account of those *frequent casualties*, which different practitioners have observed to arise from its use, and have faithfully communicated to the public. I hope he does not find a renitency in himself to consider them as *something more* than "morbid coincidencies," or "accidental occurrences," through fear of acknowledging the imperfection of his theory. As he cannot have the vanity to suppose, that his dissertation is exempt from the frailty he has in common with other men, so it would be more honourable to himself, more satisfactory to the public, and would more extensively diffuse the benefit of his discovery, if he would attempt to connect these casualties with his theory; arrange the numerous detached occurrences, to which the cooling treatment of gout has given an unexpected birth; and digest them into the body of his work. Who is likely to be so competent as himself, to elucidate this obscure subject, and to point out the particular constitutional character, where injury is to be apprehended from subjecting any part of the system to a reduced temperature? And besides, ought the public to be disappointed in their reasonable expectation, that having sanctioned, by the authority of his example and recommendation, a practice in which the welfare of thousands is involved, and defended his doctrine with a shield of science, which, however, the shaft of casualty threatens to pierce, that he would, at least, endeavour to render his pathology of the disease less vulnerable, and to place it more out of the reach of attack? Would not this be more worthy of a philosopher, than to tell us, that "he has yet to learn that the cessation of torture on the extremities," however suddenly produced by cold affusion on the affected parts (for this it must mean, if it means any thing applicable to the subject) can give rise to fatal excitement, whether inflammatory or nervous, on on the visceral organs?" (see his remarks on Mr. O'Neal's case, in the Medical and Physical Journal); or content himself with asserting his belief, that "the colouring is highly overcharged, the morbid symptoms greatly exaggerated," (see his reply to Mr. Edlin) when similar symptoms are found, in nearly the same degree, to have arisen in other similar cases, under nearly similar circumstances, and are described by persons whom he cannot object to as prejudiced against his theory; for instance, Messrs. Scott and Taynton, and Mr. O'Neal, who, on the contrary, are avowed favourers of it?

But since this task seems, at the present moment (I hope it will not continue) to be declined by Dr. Kinglake, the remarks, which the perusal of the above-mentioned communications has occasioned me to make, are intended, in some degree, though certainly in a very inadequate one, to supply that deficiency.

Having made these preliminary observations, I proceed, without further delay, to the proper subject of this paper.

The consequences of a premature and injudicious recourse to the cooling treatment of gout have been denominated *casualties;* which, however, is an objectionable name for that effect which we have reasou to expect will ensue, and which we find does in fact ensue from a given cause. For they arise naturally, as repeated experiments have shown, from the improper, not to say empirical use, or rather abuse, of one of the greatest and most invaluable remedies in gout, when exerting its influence on fit occasions. From the censure which this remark implies, although I cannot vindicate Mr. O'Neal, it will soon be evident, that Mess. Scott and Taynton are perfectly exempted; and that their confidence in the beneficial effects of reduced temperature, when cautiously administered, has indeed rescued from the imputation of inefficiency, and of injury, this noble remedy, at a time when, from the preceding failure, it was in danger of incurring that double censure; when few, indeed, of the professed admirers of Dr. Kinglake's theory would have ventured on a repetition of the refrigerant application; but which these gentlemen, guided by the enlightened view in which they contemplated the symptoms before them, did not scruple to repeat; and the sequel of the case, which was favourable, attested and confirmed their sagacity.

The case communicated by Messrs. Scott and Taynton may seem indeed, on a superficial view, to bid defiance to any attempt at explanation, that can reconcile it to either the old theory of gout, or the new one of Dr. K. Upon a nearer inspection, it will be found to favour that pathology of the disease, which, I believe, is entertained by the generality of the profession; to satisfy us that gout is *not* merely a *local* malady, but a *constitutional* affection; and to confirm the propriety of a *cautious* exhibition, but by no means of the total relinquishment, of the refrigerant remedy.

In the first attack, of October 10, 1803, the propriety of a precautionary cordial medicine is very apparent; for it may well be supposed to have preserved the patient, who had just before complained of *general indisposition*, on treating the inflamed part with cold applications, from that unusual torpor which, in a subsequent attack, when the patient himself unadvisedly made a similar application without that precaution, is said to have occurred, and to have been attended with other formidable symptoms of constitutional disturbance. The next day, when the cooling application was made to the knees, *as soon as they became painful*, and, as it should seem, before inflammation was fixed on those parts, and when no precautionary medicine was exhibited internally, the head and stomach became considerably affected. But, as the whole of this attack seems to have been a very slight one, so these affections were easily removed by some stimulating medicine; and from that moment the patient gradually and speedily recovered.

The second attack, of October 1804, appears also to have been a slight one, and to have been removed without constitutional disturbance, by the refrigerant process evincing the perfect efficiency of that remedy, under judicious management. For it is to be observed, the patient himself does not appear to have ventured this time on the cooling application, till his medical advisers assented to his wishes; which, as they lived at a distance from their patient, probably allowed time for the internal viscera to recover their accustomed vigour, and, on this account, no constitutional disturbance supervened.

But in the last and principal attack, which happened about a month afterwards, we are told, *without the least delay or hesitation*, and consequently without the direction of his medical pilots, who, on former occasions, had steered him safely through the shoals and quicksands of this distemper, he plunged into water far beyond his depth, and was certainly very near *sinking*; for he was immediately seized with an "unusual torpor over the whole frame, an intense pain in the stomach, frequent vomiting, and delirium; lirium;" and, what is truly astonishing, he continued the cold application '*two hours*, with a perseverance and courage worthy of a better cause, notwithstanding the accession of those formidable symptoms. Certainly Alexander the Great, when, bathed in sweat, he plunged into the Cydnus,* must yield the palm of superior intrepidity to Mr. Mace of Chislehurst.

We find, in the course of this last attack, that the constitutional affection was as manifest, when the extremities were covered with flannel, as when the refrigerant plan was attempted. But I should only infer from this circumstance, and it is evident from the detail, that the patient was not yet recovered from the injurious effects of the two hours exposure to the refrigerant process; and it is probable, therefore, that in so strong a predisposition to torpor, a very small exposure to any other debilitating cause, not noticed, and guarded against, might have produced both the constitutional affection, and the new inflammation of the right hand, which now occurred.

There is only one more remark, suggested to me by the concluding circumstances of this case, which demand particular attention, for the proof which they afford of the *safety* and *efficacy* of the refrigerant remedy, at *a proper period* of genuine gout, as the *necessity of caution* had been clearly demonstrated, at a preceding period of the same disorder. Notwithstanding the patient had been so great a sufferer, in the former part of this attack, from his own injudicious application of a remedy which he was unable to manage and controul, yet we find that the medical attendants were not deterred by this discouraging circumstance (and surely their confidence, under the guidance of discretion, reflects on them the highest honour), in a subsequent

---

* It is curious to observe not only *the effects on the body*, but the *time* also, which Mr. Mace's fellow-sufferer, from the same element, chose for his celebrated plunge. *Æstas* erat, et diei *fervidissimum tempus* cæperat. Pulvere ac sudore simul perfusum regem invitavit liquor fluminis, ut *calidum adhuc corpus* ablueret. Descendit in flumen. Vixque ingressi subito horrore *artus rigere* cæperunt: *pallor* deinde suffusus est, et *totum* propemodum *corpus vitalis calor* reliquit. *Expiranti similem* ministri manu excipiunt, nec satis *compotem mentis* in tabernaculum deferunt. Quintus Curtius, lib. 3. The learned reader may remember, that the river Cydnus, which was remarkable for its coldness, is celebrated by an ancient author, I believe, Vitruvius, who flourished in the Augustan Age; for what? *for curing the gout by bathing the legs in it.* I have not, at this moment, an opportunity of consulting the original :- but, in Ainsworth's Dictionary, under the proper name Cydnus, there is a reference to the passage.

part part of the same attack, when the inflammation ran high on the extremities, and when the constitutional disorder appeared to have terminated, from seizing that favourable moment, and recurring to a remedy which they knew to be the only adequate one to subdue gouty inflammation. The success was answerable to the confidence which they assumed; and exhibits the triumph of the cooling treatment of gout, when a proper degree of caution directs the application of that important remedy.

With respect to the other case, communicated by Mr. O'Neal, the *gouty* character is so strongly marked, that I think it unnecessary to attempt a refutation of the opinion, suggested by the author himself, and assented to by Dr. Kinglake, that it was, from the *commencement, an apoplectic attack.* For though gout and apoplexy have been supposed, by some authors, to be nearly allied to each other, yet their distinguishing features are so widely different, that I believe the one was never before mistaken for the other; nor apoplexy ever known to "*commence* with a torpor of the stomach, succeeded by a considerable *swelling* and *inflammation* of the foot." These particular symptoms are asserted, by Mr. O'Neal, to have characterized the *commencement* of his patient's illness; and, agreeably to the pathology which is entertained, I think, by nine-tenths of the profession, and to the description I gave of it in the 70th Number of your Journal, it is impossible for me not to recognize that distemper which is called gout.

There is reason to believe, indeed, that the *constitutional* torpor, in particular temperaments, is of so short duration, and so immediately succeeded by *topical* inflammation, as to escape the notice sometimes of the patient himself. The torpor of the case before us was of a more permanent nature; and not having been removed by the *salutary* effects on the system, which the inflammation of the foot, aided by proper medicine, or perhaps without medicine, in the space of two or three days, would probably have produced, but, as it is reasonable to suppose, *increased* and *extended* by the topical application of cold water first to the foot, then externally and internally to the region of the stomach itself, can we be surprized that the *brain* should become involved in a state of similar torpor? The pernicious influence of reduced temperature, on a habit strongly predisposed to torpor, is distinctly marked throughout the whole progress of this interesting case. An exposure to a drizzling rain all night, seems, in the first instance, to have produced a torpor on the skin, which which sympathetically affected the stomach. Then a *natural* effort was made to remove it by topical inflammation of the foot; but this being prevented by the contrary effects of a mistaken theory, the torpor appears to have been driven from the stomach to the brain. It was still pursued, when concentrated on that important organ, with fatal perseverance in the same mistaken treatment; until, at last (what other termination could be expected?) it seems to have been extended to the only remaining vital organ, where, to *stop its motions,* or to be *torpid,* is inevitable dissolution.

Mr. O'Neal, rather unseasonably, I think, at the conclusion of such an unfortunate case, declares his resolution of *following up* the same mode of treatment, until he sees *good reason* for relinquishing it. It is natural to ask him, what better reasons does he desire, or expect to find, than the result of the experiment which he has made, and most honourably permitted to be published? But it is not necessary to pursue my comments on this Case to any greater length; and I am sorry that my argument has led me to observations, which imply so much censure on the conduct of a professional man, who, I doubt not, in all other respects, is eminently useful in the station which he fills. If the liberty which I have taken should be thought to stand in need of further apology, I might defend it by the authority of one, who, on subjects relating to Ethicks, will not be found often mistaken; and apply to this particular occasion the more general sentiment of that great moralist: "He that writes, may be considered as a kind of general challenger, whom every one has a right to attack; since he quits the common rank of life, steps forward beyond the lists, and offers his merit to the public judgment. The duty of criticism is neither to depreciate nor dignify by partial representations; but to hold out the light of reason, whatever it may discover; and to promulgate the determinations of truth, whatever she shall dictate." Rambler, 93.

The observations, to which the two preceding cases of Gout have given rise, in conjunction with my remarks in the 72d Number of your Journal, on Mr. Edlin's publication, have no other object in view than to execute, as well as I was able, this *duty of criticism.* The reader is requested to consider them as supplemental to the paper on Gout, which is published in your 70th Number; as they are a continuation of that subject, and corroborative of the theory which is there submitted to the public judgment.

ment. The inferences which, I think, are fairly deducible from the whole are the following: That the reputation of the cooling practice is yet unimpugned by the accession of the formidable symptoms of the one, or the calamitous issue of the other two cases; that in all the three instances, the harm which ensued is imputable to the *injudicious* use of that remedy; that the cooling treatment should not be hazarded, till the internal viscera have discovered indubitable signs of performing with accustomed energy their proper functions; that this will *rarely* happen till the local inflammation has existed for some hours, or two or three days, or, in some cases, even a longer period; that it will happen sooner in some patients than in others, owing perhaps to some inexplicable peculiarity of temperament, or to the means which might be employed to produce that desirable effect; that when it *does* happen, no *greater degree* of cold should be employed, nor applied over a *larger surface,* nor continued for a *longer duration,* than is sufficient to subdue the local inflammation; that with these necessary precautions, no *casualty,* or *accidental occurrence,* or *morbid coincidence,* (unphilosophical terms, declarative of our ignorance) need in general to be apprehended; that if, notwithstanding every conceivable precaution, as the *stomach* in particular seems to retain a strong predisposition to torpor, owing perhaps to the want of *habit* in the new motions of health and energy, symptoms of constitutional disturbance should discover themselves,—the refrigerant application must be immediately removed, and the torpor relieved by suitable stimulants; but that, in such a case, *after sufficient delay,* the local inflammation, if it should return, is to be encountered with a repetition of the cooling process; and a happy termination be confidently anticipated.

If these conclusions are legitimately deduced, allow me to express an anxious hope that the time is not very remote, when the practice of cold affusion in Gout will be universally adopted by *discriminating* practitioners; and that unfounded prejudices will vanish before the accumulated evidence of the advantages arising from its *judicious* administration.

I am, &c.

JAMES MANTAL.

